# The association between diabetes and depressive symptoms varies by quality of diabetes care across Europe

**DOI:** 10.1093/eurpub/cky050

**Published:** 2018-04-03

**Authors:** Eva A Graham, Katie H Thomson, Clare L Bambra

**Affiliations:** 1 Department of Epidemiology, Biostatistics, and Occupational Health, Faculty of Medicine, McGill University, Montreal, QC, Canada; 2 Douglas Mental Health University Institute, Montreal, QC, Canada; 3 Institute of Health and Society, Faculty of Medical Sciences, Newcastle University, Newcastle upon Tyne, UK

## Abstract

**Background:**

Depressive symptoms are more common in adults with diabetes and may arise from the physical and psychosocial burden of disease. Better quality of diabetes care may be associated with a reduced disease burden and fewer depressive symptoms.

**Methods:**

This cross-sectional study included 34 420 participants from 19 countries in the European Social Survey Round 7 (2014–2015). Countries were grouped into quartiles based on their quality of diabetes care as measured in the Euro Diabetes Index 2014. Individual-level depressive symptoms were measured using the 8-item Center for Epidemiologic Studies—Depression Scale. Negative binomial regression was used to compare the number of depressive symptoms between adults with and without diabetes in each quartile of diabetes care quality. Analyses included adjustment for covariates and survey weights.

**Results:**

In countries with the highest quality of diabetes care, having diabetes was associated with only a 3% relative increase in depressive symptoms (95% CI 1.00–1.05). In countries in the second, third and fourth (lowest) quartiles of diabetes care quality, having diabetes was associated with a 13% (95% CI 1.08–1.17), 13% (1.08–1.19) and 22% (1.14–1.31) relative increase in depressive symptoms, respectively.

**Conclusion:**

The association between diabetes and depressive symptoms appears stronger in European countries with lower quality of diabetes care. Potential pathways for this association include the financial aspects of diabetes care, access to services and differential exposure to the social determinants of heath. Further research is needed to unpack these mechanisms and improve the quality of life of people with diabetes across Europe.

## Introduction

Diabetes is highly prevalent in European adults, with a prevalence of approximately 10.3% of men and 9.6% of women.^1^ Depression among people with diabetes is associated with poorer diabetes control and treatment adherence,^2^ increased diabetes complications,^3^ and a higher risk of mortality.^4^ Although it is generally accepted that depression or its symptoms are twice as prevalent among people with diabetes, estimates vary across Europe.^5^^,^^6^ In a UK study, 5.0% of participants with diabetes had possible cases of depression compared with 3.8% without diabetes.^7^ In a large city in the Netherlands, 29.7% of participants with diagnosed diabetes reported elevated depressive symptoms, in contrast to 19.4% of participants with normal glucose levels.^8^

The relationship between diabetes and depression may be partially attributable to the physical and psychological burden of managing diabetes and its complications.^9^ This burden may include financial difficulties due to diabetes expenses, effects of social stigma or unemployment as a result of ill health.^10–12^ People with diabetes may also experience stress from the daily psychological burden of monitoring dietary intake, physical activity levels and blood sugar levels.^9^^,^^13^ The burden of disease hypothesis is supported by cross-sectional evidence that diagnosed diabetes is more strongly associated with depression than undiagnosed diabetes.^8^^,^^14^ As well, having diagnosed diabetes is associated with an approximate 27% increased risk of new-onset depression compared with not having diabetes.^9^

Notably, the stress associated with diabetes may differ based on quality of diabetes care. Better quality of care may reduce the financial, social or psychological burden of diabetes and result in reduced depressive symptoms. However, very little evidence is available to evaluate this hypothesis. One study conducted in the USA found that less availability or affordability of diabetes medication and supplies was associated with increased diabetes-related psychological stress.^15^ Another study looked at differences in depressive symptoms between people with diabetes living on either side of the USA/Mexico border, which have differing levels of quality of health care.^16^ No differences in depressive symptoms were observed between groups.^16^ However, participants in this study were selected from a convenience sample and diabetes care quality was not measured in detail.^16^

Further cross-national research is warranted to better elucidate associations between diabetes, depression and diabetes care quality in non-USA settings using large samples and robust measures of quality of diabetes care. A large proportion of the research on depression and diabetes has been conducted in the USA and may not apply to European populations where healthcare systems generally provide more universal access, also termed higher levels of healthcare decommodification.^17^ This study aims to assess whether the association between diabetes and depressive symptoms differs by quality of diabetes care across 19 European countries. It is hypothesized that this relationship will be weaker in countries with higher standards of diabetes care.

## Methods

### Data

Data come from the European Social Survey (ESS), a repeated cross-sectional survey of social attitudes and values in 21 countries across Europe.^18^^,^^19^ This analysis used Round 7 of the ESS (ESS7), as this was the first time detailed information was collected on health conditions, lifestyle behaviours and the social determinants of health.^18^ Each country in ESS7 used random probability sampling to select a representative sample of individuals aged 15 and over in private households, though specific sampling methods varied by country.^18^ Sampling was completed between August 2014 and December 2015 and several attempts were made to contact initial non-respondents.^18^ The mean response rate was 56% across all ESS7 countries and ranged from 31% in Germany to 69% in Lithuania.^20^ This study included all adult participants (age 18+) from ESS7 countries except Estonia, as it did not collect information on depressive symptoms, and Israel, as it is not located geographically in Europe. All survey data and documentation are freely available at www.europeansocialsurvey.org.

### Measures

Self-reported depressive symptoms were measured using the 8-item Center for Epidemiologic Studies—Depression Scale (CESD-8). Depressive symptoms included feeling depressed, not feeling happy, feeling lonely, not enjoying life, feeling sad, feeling that everything was an effort, feeling that you could not get going, and sleeping problems. Participants indicated how much of the time during the past week they endorsed each symptom on a 4-point scale (never, some of the time, most of the time or all or almost all of the time). The CESD-8 supports a single factor structure,^21^ and a final depressive symptom score was created by summing participants’ responses for each symptom. Possible scores ranged from 0 (no symptoms) to 32 (all symptoms present nearly all the time).

Diabetes was measured in the ESS by asking participants whether they had or had experienced diabetes in the past 12 months. Self-reported diabetes has a high sensitivity in Europe when compared with medical records, reported medication or repeated self-report of diabetes (sensitivity = 0.79).^22^

Quality of diabetes care was measured using the Euro Diabetes Index (EDI) 2014 published by Healthcare Consumer Powerhouse.^23^ The EDI was developed by an expert panel of clinicians and academics to compare European healthcare systems and services.^23^ Indicators of quality of care included diabetes prevention initiatives, case finding and screening, range and reach of services (e.g. eye care, pharmaceuticals availability), access to treatment or care (e.g. patient education, coverage of diabetes supplies), procedures available (e.g. annual blood tests, eye exams) and diabetes outcomes.^23^ Information for each indicator was obtained from national and international surveys (e.g. WHO World Health Statistics), interviews with health care workers and public health experts, and national and regional diabetes registries, programs and reports.^23^ A total score for each country was calculated using a weighted sum of these indicators.^23^ Scores ranged from 509 in Lithuania to 946 in Sweden, with a maximum possible score of 1000.^23^ Importantly, a country’s relative position is more indicative of their quality of care than to their absolute score or ranking and therefore countries were divided into quartiles of quality of care for this analysis.^23^

Covariates included age in years, sex, education, marital status, current feeling about household income and employment (see table 1). Clinical covariates included the presence of heart/circulation problems (e.g. stroke) or high blood pressure in the past 12 months as well as body mass index (BMI) calculated from self-reported height and weight. The continuous covariates age and BMI were modelled using fractional polynomial models to account for non-linearly, which select the best higher-order terms for each covariate (e.g. age squared). Self-reported lifestyle covariates including smoking status, alcohol consumption and engagement in physical activity.

**Table 1:** Demographic, clinical and lifestyle characteristics of participants in the European social survey 7 by quartile of diabetes care quality

Quartiles of quality of diabetes care	Quartile 1 (highest)	Quartile 2	Quartile 3	Quartile 4 (lowest)
	*N* = 8719	*N* = 9362	*N* = 8211	*N* = 8128
**Countries in order of quality of diabetes care**	Sweden, Netherlands, Denmark, Switzerland, United Kingdom	Slovenia, Norway, France, Germany, Finland	Austria, Belgium, Portugal, Hungary, Spain	Ireland, Czech Republic, Poland, Lithuania
**Age mean (SD)**	47.4 (0.30)	49.2 (0.32)	47.5 (0.29)	44.4 (0.32)
**% Female**	51.6	51.9	51.9	52.4
**Marital status**				
Married/civil partnership/cohabitating	53.0	56.0	49.6	56.9
Divorced/separated/widowed	15.3	15.6	16.6	13.7
Never Married	31.7	28.4	33.8	29.4
**Main activity**				
Employed, retired, housework, other	91.3	91.1	86.9	92.3
Unemployed	4.9	6.3	10.0	6.7
Sick/disabled	3.7	2.6	3.0	0.9
**Education**				
Primary or lower secondary	30.3	30.4	55.2	35.6
Secondary	45.5	56.8	30.3	46.2
Tertiary	24.1	12.8	14.4	18.1
**Income feeling**				
Living comfortably	45.1	32.4	23.8	12.3
Coping on present income	39.6	50.9	46.7	61.5
Difficult on present income	11.8	13.5	21.7	22.3
Very difficult on present income	3.5	3.1	7.8	3.9
**BMI mean(SD)**	25.8 (0.08)	25.8 (0.09)	25.7 (0.07)	25.7 (1.00)
**% Heart/circulation problems or high blood pressure in past 12 months**	3.9	6.1	5.0	7.1
**% Diabetes in past 12 months**	5.0	6.4	5.3	4.2
**Total depressive symptoms mean(SD)**	4.9 (0.06)	5.4 (0.07)	6.1 (0.07)	5.4 (0.09)
**Smoking**				
Current	22.5	29.3	28.6	28.3
Former	25.4	23.6	19.9	18.9
Never	52.1	47.1	51.4	52.7
**Alcohol use in past 12 months**				
Never	17.3	16.1	27.6	21.7
Once a month or less	20.4	22.5	19.7	34.5
Once a week or less	31.2	34.1	26.3	31.9
Every day or several times a week	31.0	27.3	26.4	11.8
**Number of past 7 days did sports or other physical activity mean(SD)**	3.4 (0.04)	3.1 (0.05)	2.8 (0.04)	3.0 (0.05)

### Sample size

A total of 34 467 adults participated in ESS7 from the 19 studies included in this analysis. Those with a self-reported height of 110 cm or below were excluded, as this may be due to measurement error and obesity measures for these participants were extremely high (*n* = 17). Of the remaining sample, 91% had complete information on all variables. Most participants with incomplete information were missing BMI values (*n* = 1113), presence of heart/circulation problems (*n* = 594) or physical activity levels (*n* = 383). Missing demographic, clinical and lifestyle variables were imputed using ordered logistic regression (education level, satisfaction with current income, number of days of physical activity), multinomial logistic regression (sex, presence of heart problems or diabetes, marital status, smoking status, alcohol consumption) or linear regression (age, BMI) regression with all other variables as predictors. As well, approximately 2.2% of the sample had missing information on at least one depressive symptom (*n* = 752). Depressive symptoms were imputed using ordered logistic regression on all other depressive symptoms as well as the demographic and clinical characteristics listed above. Twenty complete imputed datasets were created. The final sample included34 420 participants who had complete information following multiple imputation.

### Statistical analysis

The outcome of total number of depressive symptoms was modelled using negative binomial regression in order to reflect the positively skewed distribution of depressive symptoms in the European population. Presence of diabetes, diabetes quality of care quartile and an interaction term were included as predictors. A non-zero interaction term would indicate that the association between diabetes and depression varies depending on the quartile of diabetes care quality. Sensitivity analyses examined the same associations by sex and education level. Standard error estimates incorporated clustering by country as well as variance from multiply imputed datasets. Sampling weights were used in all analyses to account for (i) selection probabilities of each participant based on each country’s sampling design and (ii) sampling error and non-response based on age, gender, education and region.^24^ Population weights were applied to account for differences in the population size of each country.^24^ All analyses were conducted using Stata version 12 and used a significance level of 0.05.

## Results

The highest quality of diabetes care was observed in Nordic countries, while lower quality of care was more prevalent in Eastern Europe (table 1). This is consistent with trends observed in overall healthcare in European countries in the same year, albeit with some discrepancies.^25^ Country rankings are also somewhat consistent with Bohm et al.’s classification of health care systems based on health care regulation, financing and service provision.^26^ Countries with National Health Service or National Health Insurance systems (healthcare regulation and financing by the state) include Denmark, Finland, Norway, Sweden, Portugal, Spain, the UK and Ireland.^26^ While the majority of these countries rank at top of quality of diabetes care, Spain, Portugal and Ireland have lower quality of diabetes care. Countries with Social Health Insurance systems, or regulation and financing by non-governmental organizations, include Austria, Germany, Switzerland and Slovenia.^26^ These countries generally had quality of care rankings in the second or third quartiles. Belgium, France, the Czech Republic, Hungary, the Netherlands and Poland have a mix of state, social and private healthcare regulation, financing and provision.^26^ Countries with this system were found in all quartiles of diabetes care. Country-specific scores are presented in Supplementary table S1.^23^

Table 1 presents differences in demographic, lifestyle and clinical factors between quartiles. The mean age of participants varied from 44.2 years (quartile 4)–49.2 years (quartile 2). There were approximately equal proportions of men and women in each quartile. Over half of participants in all quartiles were married, in a partnerships or cohabitating. Under 10% of the population in quartiles 1, 2 and 4 were unemployed or permanently sick/disabled, while nearly 13% of participants in the 3rd quartile were unemployed. Education levels were highest in the first and second quartiles and lowest in the third. Satisfaction with income followed a decreasing trend by quartile of care. Smoking trends were similar between quartiles, with 22 to 28% of the population as current smokers. Frequency of alcohol consumption decreased from higher to lower quartiles, and physical activity was highest in the first quartile and lowest in the third. BMI did not differ between quartiles. Patterns of heart or circulation problems, diabetes and mean number of depressive symptoms varied by quartile with no clear trend.

**Figure 1:**
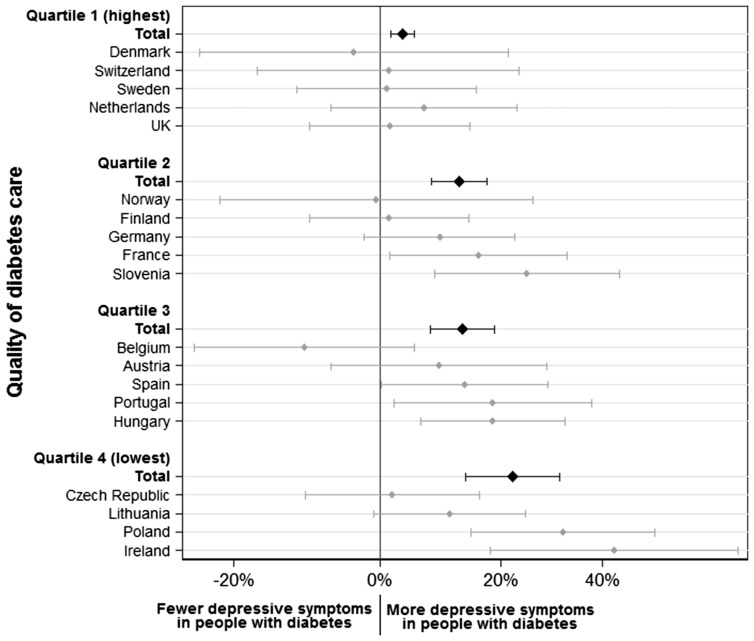
Percentage (%) increase in number of depressive symptoms in people with diabetes versus without diabetes. Estimates adjusted for age, sex, education level, marital status, current feeling about household income, employment status, presence of heart/circulation problems or high blood pressure, body mass index, smoking status, alcohol consumption and physical activity

Results from the negative binomial regression model are shown in table 2 as the ratio of depressive symptoms between people with diabetes compared with those without diabetes by quartile of diabetes care quality. Having diabetes was associated with increased depressive symptoms across all quartiles. Adjustment for demographic, clinical and lifestyle characteristics attenuated these associations, though they were still statistically significant. The association between diabetes and depressive symptoms increased as quality of diabetes care decreased. In the fully adjusted model, statistically significant differences in depressive symptoms associated with diabetes are observed between the first (highest) quartile of diabetes care and all other quartiles and between the second and fourth quartiles (figure 1).

**Table 2:** Rate ratio of diabetes on depressive symptoms by diabetes quartile

Quality of diabetes care	Model 1		Model 2		Model 3	
**Quartile 1**	1.19	1.16–1.22	1.05	1.03–1.09	1.03	1.00–1.05
**Quartile 2**	1.37^a^	1.33–1.41	1.14^a^	1.11–1.17	1.13^a^	1.08–1.17
**Quartile 3**	1.33^a^	1.25–1.42	1.16^a^	1.10–1.21	1.13^a^	1.08–1.19
**Quartile 4**	1.53^a^^,^^c^	1.36–1.71	1.26^a^^,^^b^	1.15–1.39	1.22^a^^,^^b^	1.14–1.31

Rate ratios estimated from a negative binomial regression model of diabetes, quartile of diabetes care and their interaction on number of depressive symptoms.

Model 1: unadjusted. Model 2: adjusted for individual differences in age, education, education × diabetes care quartile, satisfaction with income, gender, marital status, employment status, BMI and heart/circulation problems or high blood pressure. Model 3: additionally adjusted for smoking, alcohol use, alcohol use × diabetes care quartile and number of days doing physical activity.

a Significantly different from Quartile 1.

b Significantly different from Quartile 2.

c Significantly different from Quartile 3.

**Figure 2:**
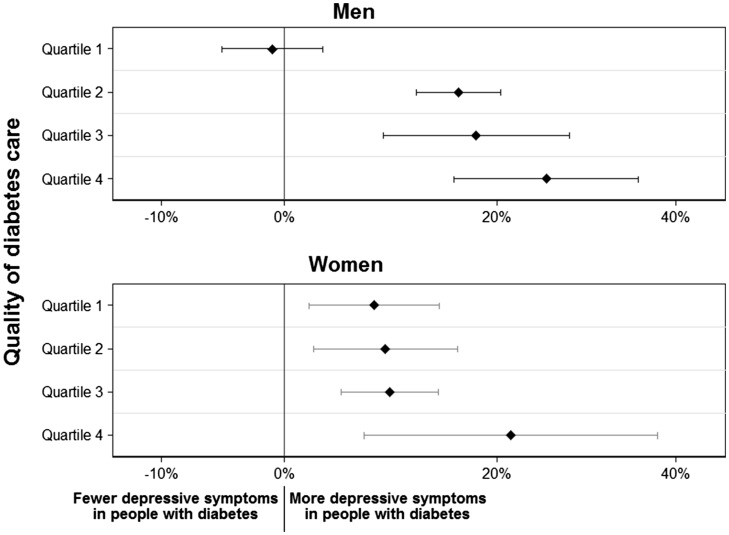
Percentage (%) increase in number of depressive symptoms in people with diabetes versus without diabetes by sex. Estimates adjusted for age, education level, marital status, current feeling about household income, employment status, presence of heart/circulation problems or high blood pressure, body mass index, smoking status, alcohol consumption and physical activity

Figure 2 presents the main analysis stratified by sex. Men with diabetes in countries in the highest quartile of diabetes care showed no increased depressive symptoms, but higher depressive symptom scores were observed in men with diabetes in the second, third and fourth quartiles. In women, diabetes was associated with increased depressive symptoms even in countries with the highest quality of diabetes care, but differences between quartiles were not statistically significant. A similar pattern of increasing depressive symptoms among people with diabetes in lower quartiles of diabetes care was observed in each level of education (Supplementary figure S1).

## Discussion

The results of this analysis suggest that diabetes is more strongly related to depressive symptoms in European countries with poorer quality of diabetes care. While diabetes was associated with an increased rate of depressive symptoms across all levels of diabetes care quality, this association was weakest in countries with the highest quality of care (RR = 1.03, 95% CI 1.00–1.05) and strongest is countries with the poorest quality of diabetes care (RR = 1.22, 95% CI 1.14–1.31). These results suggest that improved quality of diabetes care may reduce some of the psychological burden associated with living with diabetes.

These results are consistent with interventions that have reported improved depression scores in participants who received increased diabetes monitoring, education or prevention. One trial showed that people with Type 2 diabetes who used a glucose-monitoring device, kept a diary of glucose levels and intake, and received counselling experienced an improvement in depressive symptoms.^27^ Those who only received general counselling about their diet and lifestyle did not.^27^ Another trial showed that the implementation of a diabetes prevention program was associated with a decrease in depressive symptoms in the intervention group compared with the control group.^28^ The authors of both trials suggest that decreased depressive symptoms may be related to a heightened sense of control over diabetes.^27^^,^^28^ However, a meta-analysis of web-based interventions that promoted better diabetes care did not show that such interventions were associated with decreased depression.^29^

Further analyses in this study examined associations between diabetes and depressive symptoms comparing men and women. In countries with poorer quality of care, men showed a stronger association between diabetes and depression than women. This is consistent with previous meta-analyses that report sex-specific associations.^6^^,^^14^ This may be partially attributable to women’s greater use of diabetes-related healthcare services, higher adherence to glucose monitoring and selfcare, or increased social support.^30–33^ However, in countries with the highest quality of care, diabetes was not associated with increased depressive symptoms in men but there was an association in women. This may allude to an added burden of managing diabetes in addition to childcare and family responsibilities, which are largely the role of women in all European countries.^34^ Women also report more health conditions and diabetes complications than men, such as poorer physical functioning, higher cholesterol levels, and more diabetes-related complications, which may contribute to increased depressive symptoms.^30^^,^^35^ Similar to the main results, analyses stratified by educational status suggest that lower quality of diabetes care is associated with higher depressive symptoms across all educational levels. As well, higher educational levels appear to be associated with fewer depressive symptoms in diabetes in countries with higher levels of quality of care, consistent with prior research.^36^ This may be due to more positive attitudes towards diabetes and better coping strategies among those with more education.^36^

Of course, quality of care is only possible pathway linking diabetes with an increased risk of depression. Our findings might therefore also reflect wider issues within the welfare or health care systems or indeed other individual factors such as the social stigma related to diabetes. However, by controlling for lifestyle factors and socio-demographics, we hope to have taken into account some of these wider issues and isolated the role of quality of care.

### Strengths

This is the first nationally comparative study of depressive symptoms and diabetes across Europe and provides novel evidence on quality of diabetes care and psychological symptoms associated with diabetes. This analysis used a large, representative dataset of participants in 19 European countries. The ESS7 ensured representativeness though methods such as strict probability sampling and re-contacting initial non-respondents.^18^ As well, survey weighting and multiple imputation of missing variables were used in this analysis to minimize potential bias from selection of participants and non-response. Due to the number of countries included, comparisons were possible between different levels of quality of diabetes care using an objective, international measure.

### Limitations

Limitations of this study include uncertainty in the temporal order of diabetes and depressive symptoms, as the dataset was cross-sectional. Consequently, causality cannot be inferred. Unmeasured confounding may be present due to differences in mental health care across Europe. However, there are currently no tools available that allow a detailed comparative assessment of mental health care across Europe. Unmeasured confounding may also be present on an individual level from diabetes complications that were not measured the European Social Survey.^37^ This analysis was also unable to differentiate between Type 1 and Type 2 diabetes, although elevated depressive symptoms are associated with both types of diabetes in European populations.^38^

Diagnosed diabetes was assessed using self-reported measures and may have been underestimated. Yet validation studies suggest that self-reported diagnosed diabetes has high concordance with physician records or antidiabetic drug use.^39^^,^^40^ Furthermore, misclassification of diabetes would bias the association between diabetes and depressive symptoms towards the null.

Finally, quality of diabetes care may differ substantially within a country, and the measure used in this study does not capture regional or local granularity. The authors of the Euro Diabetes Index acknowledge this limitation, but suggest that this index may nonetheless be meaningful when interpreted with caution.^23^ Further research should consider more local measures of quality of diabetes care.

## Conclusions

Results suggest an inverse association between national quality of diabetes care and depressive symptoms among people with diabetes. In countries with high quality of diabetes care, diabetes was associated with a small increase in depressive symptoms while countries with poorer quality of care showed a larger increase in depressive symptoms among people with diabetes. This association persisted when adjusted for individual demographic, clinical and behavioural characteristics. Quality of diabetes care may therefore be important not only for physical complications of diabetes but also for depressive symptoms and psychosocial outcomes.

## Funding

EG received a scholarship to complete this research from the Queen Elizabeth Scholars Program, funded by Universities Canada, Community Foundations of Canada and the Rideau Hall Foundation, in collaboration with the Institute for Health and Social Policy, McGill University. CB and KT are part of the HiNEWS project—Health Inequalities in European Welfare States—funded by NORFACE (New Opportunities for Research Funding Agency Cooperation in Europe) Welfare State Futures programme (grant reference: 462-14-110). For more details on NORFACE, see http://www.norface.net/11. CB is a member of Fuse, the Centre for Translational Research in Public Health. Funding for Fuse comes from the British Heart Foundation, Cancer Research UK, Economic and Social Research Council, Medical Research Council, the National Institute for Health Research, under the auspices of the UK Clinical Research Collaboration, and is gratefully acknowledged. The views expressed in this paper do not necessarily represent those of the funders or UKCRC. The funders had no role in study design, data collection and analysis, decision to publish or preparation of the manuscript.

*Conflicts of interest*: None declared.

**Key points**

- Diabetes is associated with increased depressive symptoms across Europe
- An increased association between diabetes and depressive symptoms was observed in countries with lower quality of diabetes care compared with those with higher quality of diabetes care
- Higher quality of diabetes care may reduce the burden or stress of disease and is associated with fewer depressive symptoms among adults with diabetes

## Supplementary Material

Supplementary Figure S1

Supplementary Table S1
