# Supplementary figures and images for: The association between diabetes and depressive symptoms varies by quality of diabetes care across Europe
Source: Eur J Public Health. 2018 Apr 3;28(5):872–8. doi: 10.1093/eurpub/cky050 (PMC6148969; doi:10.1093/eurpub/cky050)

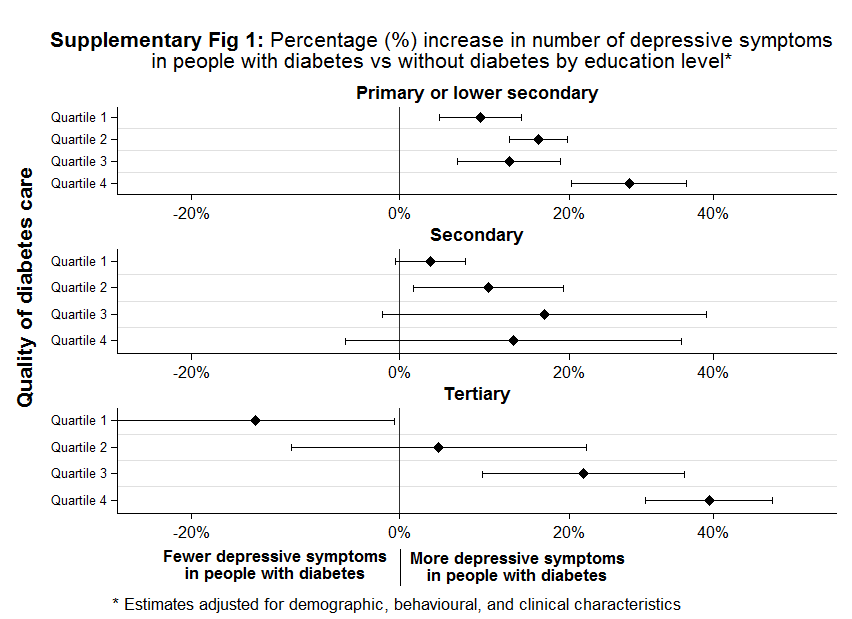

Supplement: Supplementary Figure S1 [file cky050_figure_s1.png]
