# Supplementary material for: The association between diabetes and depressive symptoms varies by quality of diabetes care across Europe
Source: Eur J Public Health. 2018 Apr 3;28(5):872–8. doi: 10.1093/eurpub/cky050 (PMC6148969; doi:10.1093/eurpub/cky050)
Supplement: Supplementary Table S1 [file cky050_table_s1.docx]

**Supplementary Table 1:** Euro Diabetes Index 2014 Total Scores^a^ by Country and Quartile

| **Quartile** | **Country** | **EDI total score** |
| --- | --- | --- |
| 1 | Sweden | 936 |
|  | Netherlands | 922 |
|  | Denmark | 863 |
|  | United Kingdom | 812 |
|  | Switzerland | 799 |
|  | **Mean** | 866 |
| 2 | Slovenia | 778 |
|  | Norway | 746 |
|  | France | 736 |
|  | Germany | 733 |
|  | Finland | 715 |
|  | **Mean** | 742 |
| 3 | Austria | 706 |
|  | Belgium | 700 |
|  | Portugal | 668 |
|  | Hungary | 651 |
|  | Spain | 633 |
|  | **Mean** | 672 |
| 4 | Ireland | 612 |
|  | Czech Republic | 597 |
|  | Poland | 564 |
|  | Lithuania | 509 |
|  | **Mean** | 571 |

a: Source: Cebolla Garrofé B, Björnberg A, Yung Phang A. Euro Diabetes Index. Health Consumer Powerhouse, 2014. Available from: http://www.healthpowerhouse.com/files/EDI-2014/EDI-2014-report.pdf.
